# On the front line: Health professionals and system preparedness for Zika virus in Peru

**DOI:** 10.1002/ijgo.13047

**Published:** 2020-01-23

**Authors:** Ruth Iguiñiz‐Romero, Lucia Guerra‐Reyes

**Affiliations:** ^1^ School of Public Health and Administration Cayetano Heredia University Lima Peru; ^2^ School of Public Health Indiana University Bloomington IN USA

**Keywords:** Congenital Zika syndrome, Health personnel, Peru, Surveillance, Zika virus

## Abstract

**Objectives:**

To analyze the initial healthcare response to the Zika virus in Piura, Peru, and assess the perceptions of midwives and nurses regarding their role in prevention of Zika virus and management of congenital Zika syndrome (CZS).

**Methods:**

This ethnographic study used a rapid qualitative assessment design. Data were collected through a focus group with midwives and in‐depth interviews with midwives (n=11) and nurses (n=5).

**Results:**

The focus of the early Zika virus response in Piura was on pregnant women and vector control. Midwives received some training on Zika‐related care during the early response. Nurses did not receive any Zika‐specific training. Neither nurses nor midwives were trained in neonatal CZS surveillance. Midwives were clear about the value and feasibility of incorporating Zika virus surveillance in their daily work, however nurses were not. They referred to lack of training and appropriate tools as limitations. Confusion about Zika virus and CZS symptomatology and effects persisted in both groups. Concerns about their own personal risk influenced the ways they engaged with Zika virus prevention in the community.

**Conclusion:**

Long‐term management of endemic Zika virus in Piura will require the engagement of both nurses and midwives as primary care providers.

## Abstract 2

A cost‐effective and culturally competent approach should include a broader focus on family planning and child development surveillance. This requires cross‐disciplinary collaboration between nurses and midwives.

## 1 INTRODUCTION

Health providers are the first line of care and surveillance for the long‐term management of any endemic disease, yet little is known about how they process and view their roles in cases of emergent and complex diseases. Zika virus is one such disease. Primarily transmitted through the bite of an infected *Aedes aegypti* mosquito, it is also transmitted through sexual intercourse, from mother to child, and through blood transfusion. Zika virus infection during pregnancy has been linked to stillbirths and to congenital Zika syndrome (CZS) characterized by varied levels of cognitive impairment, microcephaly, and a host of vision, hearing, and motor impairments. Additionally, Zika virus infection in nonpregnant women has been linked to an increased incidence of Guillain‐Barré syndrome.^1^

The characteristics of Zika virus infection present new challenges for the public health response and long‐term prevention. A large number of asymptomatic cases,^2^ the persistence of the virus in semen,^3^, ^4^ and the preference of the mosquito for living inside homes mean a higher risk of infection in low‐income urban communities and especially among vulnerable women and children.^5^ Engaging health professionals effectively in a coordinated response is key to managing Zika virus in its new endemic form.

Between 2016 and 2017 around 6000 cases of Zika virus were reported in Peru, with higher concentrations in the regions of Loreto, Piura, Ica, and Cajamarca.^6^ The number of suspected cases was much higher than the final confirmed cases; however, diagnosis was hampered by the need to send blood samples to Lima for confirmation. While a cluster of Guillain Barré cases in 2018 was suspected to be linked to Zika virus, there have been no large‐scale reports of adverse Zika virus‐related effects in Peru. However, since surveillance for microcephaly and other fetal abnormalities is limited, it is likely that cases of CZS are underreported.

In Piura, there were around 300 confirmed cases of Zika virus infection between 2017 and 2018, and 88 so far in 2019.^6^ Zika surveillance was affected by flooding due to heavy rains in February 2017, which damaged rural health posts and overwhelmed the emergency response. After the flooding a dengue epidemic swept through the city, infecting at least 44 000^7^ and killing 41 people.^8^ In the wake of these overlapping emergencies, Zika virus was low on the priorities of the regional surveillance apparatus; thus, it is difficult to ascertain the real effects of the virus. However, it is expected that Zika will follow a pattern of periodic activity similar to dengue, making it a future priority for local public health officials.

The aim of the present study was to analyze the initial healthcare response to the Zika virus epidemic in Piura and assess the perceptions of frontline personnel—midwives and nurses—regarding their role in prevention of Zika virus and management of congenital Zika syndrome (CZS).

## 2 MATERIALS AND METHODS

The data reported here were part of a larger study that included focus groups and interviews with local men and women, and health providers. The present article reports only health provider data collected in two phases: phase 1, August–September 2017; and phase 2, January–February 2018.

This ethnographic study used a rapid qualitative inquiry design, featuring intense but shorter data collection than traditional anthropological ethnography.^9^ The study site was chosen in collaboration with the Piura Regional Health Directorate.

The location of the study, the Catacaos Micro‐Network, is part of the Lower Piura Health Network. The micro‐network is comprised of an urban clinic that includes all primary care, pregnancy, birth care, and surgery facilities, and six dependent health centers, with lower levels of care, located in outlying and/or rural areas. Names of participants and their health centers have been omitted in accordance with Institutional Review Board recommendations.

**Figure 1:**
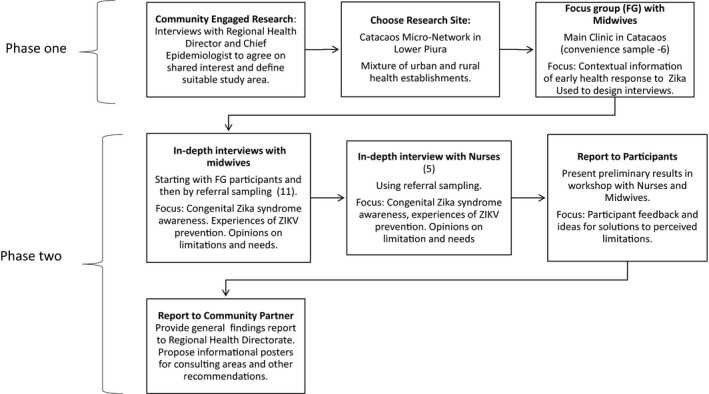
Study data collection and community engagement flowchart.

A summary flowchart of the study is given in Figure 1. The original study design only included midwives as participants. During the focus group phase, it became apparent that nurses were also important to meet the objectives of the study and they were subsequently recruited for in‐depth interviews. Data were collected through a focus group with midwives (n=6), and in‐depth interviews with midwives (n=11) and nurses (n=5). The focus group was a convenience sample of Catacaos midwives. Participants were invited in person. In‐depth interviews were recruited among focus group participants who said they were interested, and by referral, asking each participant to recommend a colleague from the micro‐network for interview. Interviewees were approached in person and via phone messaging app by one of the authors (RI). Health personnel who declined participation gave time constraints as the reason. There was no prior relationship with the specific participants, but the university of one of the authors (RI) has ongoing collaborations with the regional health directorate.

The focus group questionnaire was generated to understand the context of Zika virus interventions and served to create an in‐depth interview guide. The focus group was conducted in a meeting room at the Catacaos clinic and lasted approximately 90 minutes. Interviews asked about initial Zika response, knowledge of Zika, perceptions about clinic‐based Zika virus prevention, and perceptions of how Zika virus has affected the midwives’ and nurses’ personal and professional lives. Interviews took place where participants felt comfortable—most in their workplace after hours and some in a nearby cafeteria. Each interview took 45–70 minutes. All participants provided verbal consent and received a study information sheet in Spanish. Field notes were taken (RI) during the interviews to register main ideas and contextual information. No other people were present during data collection. Recruitment for interviews continued until saturation was reached.

**Figure 2:**
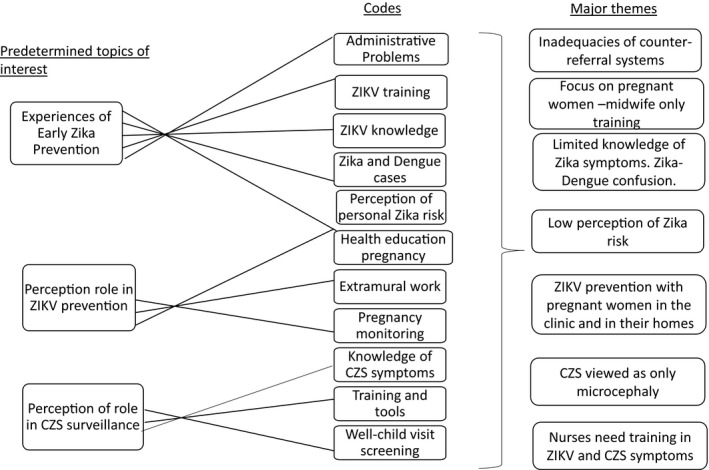
Coding tree.

Data were recorded, de‐identified, and transcribed verbatim. Transcriptions were coded deductively for predetermined and emergent codes using ATLAS‐ti version 8 (ATLAS.ti Scientific Software Development GmbH, Berlin, Germany). Focus group data served to understand the context and create a semistructured interview guide. All data presented here are from in‐depth interviews. The authors coded independently, and coding structure was discussed and harmonized at two points during the process. Codes were organized into categories in response to research questions. Major themes in each category were derived from the data and represent shared experiences (Fig. 2). Transcripts were not returned to participants, and no interviews were repeated. All participants were invited to a workshop where they provided feedback on preliminary results.

The study was approved by the Pan‐American Health Organization Ethics Review Board and the Ethics Committee of Peruana Cayetano Heredia University in Lima, Peru. The protocols and informed consent forms were shared with regional partners and assessed for cultural concordance by local research team members.

## 3 RESULTS

All midwives and nurses in the study were female, with an average age of 42 years. The average age of the midwives was 47 years, whereas for nurses it was 34 years. Of the 17 midwives and 5 nurses, 14 were partnered and 14 had children. Most had worked in the Catacaos health micro‐network for between 1 and 25 years.

Midwives and nurses have clearly delimited professional responsibilities in urban, well‐staffed facilities, like the Catacaos clinic. Midwives oversee sexual health, family planning, and prenatal and obstetric care. Nurses handle neonatal and primary pediatric care. However, at rural facilities, often one nurse or midwife was in‐charge of providing all care to women and children.

### 3.1 Experiences of initial response to Zika prevention in Piura

The increased risk posed by Zika during pregnancy meant that the initial response involved midwives directly, but not nurses. During early Zika virus interventions (May 2016 to May 2017) most midwives interviewed received training in Zika virus symptoms and counseling, and received mosquito nets, bug repellent, and condoms for their pregnant patients. Local flooding followed by an outbreak of dengue in 2017 disrupted Zika virus surveillance and repurposed all personnel to the dengue response. However, features of this early training were incorporated into midwives’ ongoing pregnancy care protocols. As M11 stated, midwives were trained to provide this information in the first prenatal visit:

The patient comes to her first control; everything is explained to her, we give her advice […] she has to dress up, at night even though is hot, avoid being bitten by the mosquito, put on her mosquito net, use the repellent, have sex with the husband with a condom. Midwife 11

Nurses did not participate in early Zika virus training. Interviewees anecdotally described how they learned about Zika virus through the media and colleagues. They assumed that they would likely be involved only if any cases of microcephaly occurred. However, even in cases of suspected microcephaly, care was derived to specialists, and primary care personnel lost track of cases:

The girl, the girl's growth, the head circumference was not improving. She was only one month old […] The [doctor] saw her and they sent her to the lab and there I think they confirmed something, or maybe not and then the mother was sent to gynecology. From there I don't know, I lost track and did not follow up. Nurse 4

### 3.2 Confusion and misunderstanding about Zika virus transmission and symptoms

Interviewees knew that Zika virus is transmitted by the *Aedes aegypti* mosquito, like dengue. However, it was frequently confused with dengue. For example, M6 reported:

Zika is another type of dengue, more aggressive and what's worse it attacks women in the first trimester and produces microcephaly. Midwife 6

Similarly, M7 said that the symptoms are:

[…] fever, increase temperature, joint pain. But only the lab can tell us the difference with dengue […] because it is the same symptoms, almost. Though maybe that [gestures towards arm] rash, although you get that with dengue too, so only the lab will tell you. Midwife 7

Some midwives, those working in the urban clinic, had received more training and could identify skin rashes as a key symptom of potential cases to differentiate Zika virus from dengue:

Well, Zika is a disease transmitted by the same mosquito that transmits dengue, yellow fever and chikungunya. In a pregnant woman it is very dangerous because it directly affects the baby. What the virus does to the baby, it eats the brain. That's what I say to them. It destroys the brain, and the child can be born without the ability to see, it doesn't hear, doesn't talk, doesn't walk. And it also depends on the gestational age when it is affected. That it is very important that they take care to not get bitten by a mosquito, because we are in an endemic area, because we had had many cases of dengue and because that is the same mosquito that transmits Zika, then we are not free [of risk]. The other thing that is very worrisome is that it is a virus that doesn't produce many symptoms, maybe women will feel a slightly higher temperature that can be dismissed, but that something that can indicate [Zika] is the appearance of skin rashes. That is what we tell them. Midwife 8

However, no midwife indicated that rash on the palms is one of the best ways to distinguish symptomatic Zika virus infection. It is encouraging that almost all midwives did identify the sexual transmission of Zika virus.

Nurses had little knowledge about Zika virus; they only identified mosquito‐borne transmission of the virus and did not mention any other forms of transmission. They had not received any specific training on Zika virus, yet some had found information from other sources. For example:

I know that Zika is viral, the same vector as dengue and chikungunya. All the Americas are endemic. Pregnant women are more vulnerable and the child is born with microcephaly, so the damage when infected is really severe. [Where did you get that information?] From the internet mainly. I like to know how new things will affect us. Nurse 4

Nurses knew very little or nothing about CZS, with the exception of the presence of microcephaly. There had been few cases of Zika virus, and these had been managed by medical doctors, which may have impacted the ability of nurses to identify symptoms, for example:

As far as I know, no Zika has arrived here, [mainly] because it's the doctor who monitors things [like] high fever I haven't touched any Zika cases so far, none, or dengue either. [Symptoms of Zika are] bone pain, nausea, vomiting I think, I don't remember the symptoms of Zika very well, just something, not very well, I would be lying. Nurse 2

Nurses’ lack of knowledge of Zika virus and CZS symptomatology is concerning given that they are often the only primary care professional in lower‐level health posts in rural areas, and because they are in charge of caring for neonates and infants. Interestingly, neither nurses nor midwives discussed Guillain‐Barré syndrome as an effect of Zika virus.

### 3.3 Referral and counter referral system limits engagement with Zika virus prevention and care

Primary care personnel are necessary for vector‐borne disease surveillance including Zika virus. Given Piura's long experience with dengue and malaria, there is an acute awareness of the importance of identifying febrile cases. Currently the same strict protocol is used to identify potential Zika virus infection cases, despite the high level of asymptomatic cases and the lower likelihood of high fever. This protocol relies heavily on medical doctors for diagnosis and referral:

So, then we work according to the ‘febrile patient’ protocol […] In triage we capture the symptomatic patient or the febrile ones. If a febrile patient presents in triage the technician will immediately ask “Where were you in the past few days?” And according to that, if there are any other associated symptoms, like joint pain, red eyes—that would be conjunctivitis—we then fill out an epidemiological form according to the dengue protocol. We are more careful if the patient is a female in reproductive age and even more if she is pregnant […] because we know that Zika is transmitted through sexual intercourse and [we take a lot of care of] the baby. Because of congenital malformations. Then according to that personnel derive the patient to the physician. Nurse 1

Rural health centers generally do not have a permanent medical doctor and refer patients to the main clinic or “head of the network” for basic triage and diagnosis. At least three interviewees noted that the physician in their rural clinic was a SERUMS—a recent medical graduate completing their rural and peri‐urban practice year. These doctors have limited experience, work limited hours and for only one year, thus they are not fully part of the rural healthcare team. For example:

The issue is that this is a level 2 [low level rural] clinic, we have a SERUMS doctor they see patients only in the morning or the afternoon. So then the nurse [me] has to step in. Nurse 1

Once referral procedures take place, patients rarely return to the primary care facility with a clear diagnosis or instructions to allow follow‐up from nurses and midwives. Many patients don't remember or understand what the outcome of the referral was, and the primary care personnel do not receive any counter referral indications:

Right now, I have a case of a patient, exact same as another, with malformations, there's a sonogram that shows malformations, I'm just about to notify epidemiology. But I don't have any confirmation. I only have those two cases, but in sonograms there are malformations that lead us to assume [that maybe there's more]. We would need to do a study to confirm. Here we notify epidemiology and nursing, and then epidemiology takes over coordinating with the Santa Rosa Hospital. […] They should return with a counter‐referral form. But, I'll be honest, that never happens. They receive care over there [hospital] and we maybe do a little follow‐up. Often patients don't tell you anything because it's all medical terminology, [they just say] “they told me it [baby] was fine.” Midwife 10

In this case and that of Nurse 1, health personnel expressed exasperation and confusion as to their current role in Zika virus care. The lack of an efficient and comprehensive referral and counter‐referral system is perceived as a barrier to effectively engage in Zika‐related care.

### 3.4 Perceptions of personal Zika virus risk is low

One key issue is the relationship of health personnel with the community and their own risk awareness. Most midwives and nurses live near where they work, they are exposed to the same risks and share similar cultural perspectives as their patients. Many are concerned about their own well‐being and care. For example, discussing their own constant vigilance for mosquito bites:

[we are concerned] as you see, here is our repellent, every time we come here [clinic] the first thing we do is zzzzzzz [apply it], the first thing we do. It is the most common measure that we all do here, not only me, we all have our repellent. As for food, we have also told everyone to drink acidic juices. Here [I have] orange, lemon, we try to drink [these] citrics [to remain healthy]. Midwife 6

However, most of these precautions center around concern for dengue. During the floods of 2017, almost a third of interviewees and many of their family members became ill with dengue. They attributed this contagion to outreach work in flooded rural communities:

Yes indeed, because we do outreach work. With the flood and everything, we did not have the Health Center, it did not work because it was flooded, and everything was spoiled. So, we have to go out to the campaign, go out to the shanty towns that belong to a rural community and there were a lot of mosquitoes, with the water and everything. And yes, we were very worried; at least we protected ourselves, we came with a long sweater, we used our repellent but still there is always the worry and we got it, we got dengue no matter how much we used the repellent, no matter how much we used the mosquito net. We got dengue anyway because the mosquito is there and there were plenty mosquitoes, there were a lot of Aedes. Midwife 11

Concern over the Zika virus contagion was varied among nurses and midwives. Those who were past reproductive age or were sterilized were not concerned for themselves, but for their daughters or other family members:

I can't have any more children even if I wanted to [sterilized] but it does worry me for my children who are young, thirteen and fifteen. Midwife 7

I am 48 my husband is 52 I am not concerned for myself; we are not getting pregnant again. But I have two nieces who were pregnant. I was concerned for them, they live in [rural areas] and they had many mosquitoes, but thankfully everything turned out ok. Midwife 8

Others conflating dengue and Zika were concerned about it because they believed that: “*it is a disease that can lead to death*.” Nurse 3

Overall, those that were still of reproductive age echoed patient attitudes and did not take any extra precautions to avoid pregnancy:

Well it hasn't meant many changes in my life really, because we are already taking preventative measures for dengue. The only thing is that I'm not pregnant, my husband uses condoms. […] we use rhythm method and condoms only on fertile days. Midwife 5

Some reported that already having a secure contraceptive method, like the implant or the shot, gave them a sense of calm since they were already avoiding pregnancy:

I would be worried if I was pregnant. I am more concerned about dengue. When we go to community outreach, we use insect repellent. […] I use the monthly shot and sometimes condoms [so Zika is not a concern]. Midwife 2

Overall, perceptions of risk among participants demonstrated little concern for Zika virus infection, though general concern for mosquito‐borne diseases, and dengue specifically, was very high.

### 3.5 Perceived role in future Zika virus prevention

Midwives had a clear perception of their responsibility in Zika virus prevention since it is part of their protocols for prenatal counseling. However, they mainly see Zika virus as related to the care of pregnancy and not necessarily to family planning or reproductive counseling:

Here we focus on pregnant women. […] But us, as midwives, we focus on our pregnant women. We prevent the same as dengue, we talk about how they could contract dengue from the mosquito bite, but also through sexual intercourse. We gave them, talked about prevention with them regarding their protection, first with insect repellent, with mosquito nets, closing doors, avoiding standing water. Midwife 7

This midwife echoed the need to protect pregnancies, and interestingly also conflates dengue and Zika.

Midwives recognize the need to extend prevention messages to nonpregnant women. However, many of these initiatives were bundled into the educational outreach related to the dengue outbreak, and they focused mostly on pregnancy care, and vector control:

Yes, [we have done] outreach to schools, for teenagers and then children, mothers clubs [the message is] to get rid of mosquitos, get rid of Zika. Midwife 6

While condom use was mentioned, it is clear that midwives mostly considered it only as part of the messages for pregnant women, not part of a general pregnancy delay strategy:

[the main messages] are for our pregnant patients definitely, and it's malformations. We want healthy babies, so they have to use all the ways to prevent it and take care. [we tell them] how they must keep water reservoirs, to use mosquito nets, bug repellent, and condoms. Midwife 4

While contraceptive counseling and promotion of family planning methods are a central part of the midwives’ work in the community, none mentioned them as a part of Zika virus prevention.

Nurses, however, did not directly consider Zika virus prevention as a direct part of their responsibility, a perception reinforced by their lack of training:

The truth, since you asked, we have not done anything. The truth, it would be good to do something, we have not thought about it. We have seen it as something that is real, of course, but not enough to give information or give informative talks to our mothers. We had not contemplated it, to be honest. But right now, we talk about it like that, we break it down, and you're right. We could start with one more topic, such as breastfeeding, child's care, caring for this [Zika virus]. Nurse 5

Those that had did make efforts to discuss Zika virus felt most comfortable talking about preventing mosquito bites and managing vector control:

Well, we haven't actually seen any Zika in children, mostly in pregnant women. But we do recommend hand washing in all office visits. Hand washing and changing water in their reservoirs at home [so it's not standing for long] and we tell them about the symptoms and such, and to go to emergency care at the health center. Nurse 3

Nurses see more of a role for themselves in CZS surveillance than in working with pregnant women.

### 3.6 Perception that Zika virus prevention should include outreach and community work

Both nurses and midwives considered that community work should be a part of future Zika virus prevention. They are already engaged in what they call “extramural” work, especially in rural and peri‐urban areas where access to health care is limited:

So, we would go out to the streets looking for pregnant women, those in their early pregnancy, which is when the effect [of Zika virus] on the product is greater. We would give them counseling, and we would also give them condoms; because of course sex is still happening, but you have to avoid it, the infection. Many of the husbands of the women work outside the community, maybe in Tumbes, or on the border, and there were foci [of Zika virus infection] in those areas. So, to avoid that after counseling we gave them condoms. We haven't had any cases so far here in this area [small rural hamlet], but we have worked really hard. Midwife 11

Health professionals advocated including Zika virus as part of ongoing community visits that all clinic personnel have to fulfill. This was piloted when Zika virus cases first appeared and could continue:

So that's how it was, we worked with pregnant women, with the general population. We do the “ten‐minute intervention.” [Health professionals] go to each house for ten minutes, to check for discarded materials, check for mosquito breeding areas. They [the community] save water because here we don't have water all day. So, there are checks to see whether those are appropriate storage for water, if they have a lid, if there are larvae, if there are flower vases with larvae, all of that. And they [the community] receive explanations, that you have to throw those away, that it's better to have your flowers but with wet earth and all of that, to avoid mosquito propagation. We also tell them to throw away all trash materials because that's where the mosquito lives. Midwife 11

Midwives also wanted to establish a way to have male partners participate in prenatal visits. Some do participate and midwives reported that this is an excellent space to engage men in discussions of sexual transmission of Zika virus, the need for condom use during pregnancy, and future contraceptive usage.

When they come to the prenatal control that makes things easier for when she comes in for her method because she already talked to him, and he already hears about it. When he comes, in some cases husbands come, we take advantage and we talk about everything because it's an opportunity. We think [it has to be now because] “When will he come back? IF he comes back.” And I'm very happy because there's many that come, maybe late in the day or on their off days from work they come with their wives. Midwife 4

### 3.7 Perceived role in congenital Zika syndrome surveillance

As explained in the referral system section, the lack of counter‐referral protocols limits the ability of midwives and nurses to follow‐up suspected Zika virus cases. This poses problems for effective CZS surveillance. While nurses see a greater role for themselves in managing and treating the effects of Zika virus in children, they have not been trained to do it:

So, there was some training, but really who got it, the midwives! We got some leaflets and condoms. Nurse 5

What I think is that [midwives] who have been trained should do replicas to all others. Here we didn't do it. They shared the materials but the proper training we did not get. Nurse 4

Nurses’ current lack of knowledge about CZS symptoms limits their ability to identify cases of CZS. Currently, most only identify microcephaly as a sign of the syndrome and focus only on head circumference. Even though they evaluate other developmental milestones, none mentioned other CZS symptoms:

For newborns, it's four visits the first month. One each week. And there we observe, we measure head circumference, abdominal, thoracic, we evaluate the child completely. Up until two months. Until the second month we measure head circumference. Nurse 3

However, proper training and tools to appropriately measure head circumference are also needed:

Something we have reinstated a lot since last year is the head circumference measurement. When I returned, I came in November 2016, I didn't see that it was done in our well‐child care. I insisted with the person in charge at the time, that it was a very important step in the child's evaluation, that we could not avoid it. They argued that there was no proper measuring tape for true head circumference measurement, the one that comes from the Ministry [of health], there wasn't even a simple measuring tape, but I thought there were ways to get it. Then we got one and from there we have been doing it, it's one of the things we do to avoid [Zika], to go on measuring, comparing with the baseline of head circumference at birth, with its age and to compare with the growth charts and see if it is in normal range. Nurse 5

In addition to pointing out limitations of the tools to monitor development, this nurse also explained measuring and identifying problems in head circumference growth as mechanisms to “avoid” Zika or its effects in children. It further reflects the existing misunderstanding about CZS, the impact of Zika virus, and opens up a discussion about the value of interventions to prevent and cure health problems over those of monitoring, identifying, and dealing with permanent/chronic health conditions.

When asked about their capacities to include any screening for neurological and cognitive problems as part of child growth and development care, nurses only mentioned one test they apply to children for psychomotor development (TEPSI) between 2 and 5 years.

What we do is TEPSI, which is a cognitive test. We do it with children who are two, three years old. We have a little book where they can identify animals, we ask them their name, how old they are. Nurse 3

Overall, nurses would like to receive more training about Zika virus, but mainly see a role for themselves in CZS management.

## 4 DISCUSSION

Effective long‐term prevention of Zika virus, including management of CZS, poses challenges for health policy and prevention strategies. Moving from early response to Zika virus infection to endemic scenarios will require coordinated policies that engage all primary care personnel.^10^

Studies have shown that health personnel in Zika endemic areas have a high level of awareness, but a low level of knowledge and preventive practices related to Zika virus prevention^11^, ^12^; especially as it relates to sexual transmission and prevention of sexual transmission.^13^, ^14^ Our results similarly demonstrate that frontline healthcare personnel in Piura (nurses and midwives) generally know about Zika virus but have received uneven training. While it is encouraging that midwives have received enhanced training, it is equally concerning that nurses have not; especially given that they also manage pregnancy care in rural and remote areas. On the other hand, sexual transmission of Zika virus is well known and emphasis on condom use during pregnancy is a central part of preventive counseling.

As in many countries, the focus of early Zika virus prevention was pregnant women. Midwives have easily incorporated and maintained Zika virus related counseling into their prenatal care work but have yet to incorporate it as part family planning and contraceptive care. Despite concerns about fetal effects, both nurses and midwives have limited knowledge about CZS. They only associate it with microcephaly, although the full spectrum of CZS includes epilepsy, cerebral palsy, delayed development of language and/or motor skills, strabismus, visual abnormalities, and heart, renal, and urinary tract disorders, among others.^15^, ^16^, ^17^

Midwives and nurses pointed to the absence of microcephaly as a mark of the relatively benign nature of Zika virus infection in Piura, especially compared with the deaths caused by dengue. However, the absence of microcephaly cases in official records does not necessarily mean that there are no cases, especially with the technical limitations described above and in the literature.^3^, ^17^, ^18^ In this region, where 10%–13% of children weigh less than 2500 g at birth,^19^ 34.3% of children under 5 years old suffer from chronic malnutrition, and 26.5% of rural children suffer from anemia,^20^ it is not unexpected nor uncommon to find newborns who are small for gestational age; thus, potentially obscuring cases based on head circumference alone. Additionally, lack of training and awareness about ZCS among nurses and midwives who interact directly with newborns also limits the possibilities of identifying other cases within the spectrum.

Women of reproductive age, who should be well aware of the risks posed by Zika virus when considering their family planning options, have not been consistently targeted either. This reflects the limitation of the primary health system to reach women of reproductive age before they are pregnant and access prenatal visits. This also reflects the political reticence around openly discussing and fulfilling women's sexual and reproductive rights and needs, including offering culturally acceptable contraception.^21^

The siloed organization of health programs and targeted populations has also been challenged during the Zika virus epidemic. The few suspected cases of newborns with anomalies required the exchange of medical information between nurses, midwives, and doctors at the primary care level, prior to their referral for confirmation, and between different levels of care, which was cumbersome, slow, and inconsistent. Lack of a counter‐referral system in place poses a challenge to children's health and CZS surveillance at primary care level.^22^

The occurrence of CZS also revealed the conditions and limitations in which standard child growth and development services are provided, and the challenges they present to identify and care for the wider spectrum of symptoms and conditions that might not present immediately or at birth. Constant follow‐up is crucial for early identification of suspected cases and remedial actions at the primary care level provided by local nurses familiar with the child during his/her first years of life.

Finally, it is important to acknowledge that health personnel at primary care centers share cultural values and practices and risk perceptions with the community they are part of and the population they serve. Their relatively low perception of Zika virus risk may reflect and reinforce local attitudes.

The present study is novel in its approach to trying to understand how health providers view themselves within Zika virus related care. However, it has some intrinsic limitations. Firstly, flooding and a dengue epidemic disrupted data collection and moved all local resources toward dengue prevention. This may have influenced the conflation between dengue and Zika virus symptoms in the area. Additionally, this was a qualitative, small sample study that may inform future research and questions but is not meant to be generalized.

In conclusion, in an endemic scenario of long‐term surveillance and management of Zika virus and CZS, diagnostic tools, training, and surveillance resources need to be increased to be effective. A cost‐effective and culturally competent approach should likely include a broader focus on family planning and child development surveillance. This supposes cross‐disciplinary collaboration between nurses and midwives. The present findings are important for health policymakers who have to design training, surveillance, and referral processes at regional and local levels.

## AUTHORS CONTRIBUTIONS

RI and LG conceived and designed the study, conducted data analysis, and drafted the manuscript. RI conducted the interviews and fieldwork. All authors contributed to the revision of the article and approved the final version of the manuscript.

## CONFLICTS OF INTEREST

The authors have no conflicts of interest.

## References

[1] Petersen LR , Jamieson DJ , Powers AM , Honein MA . Zika virus. N Engl J Med. 2016;374:1552–1563. 27028561 10.1056/NEJMra1602113

[2] World Health Organization . Zika virus factsheet. [Online]. http://www.who.int/mediacentre/factsheets/zika/en/. Accessed May 19, 2019.

[3] Allard A , Althouse BM , Hébert‐Dufresne L , Scarpino SV . The risk of sustained sexual transmission of Zika is underestimated. PLoS Pathog. 2017;13:e1006633. 28934370 10.1371/journal.ppat.1006633 PMC5626499

[4] Kim CR , Counotte M , Bernstein K , et al. Investigating the sexual transmission of Zika virus. Lancet Glob Health. 2018;6:e24–e25. 29241605 10.1016/S2214-109X(17)30419-9 PMC6713899

[5] Jamrozik E , Selgelid JM . Ethics, health policy, and Zika: From emergency to global epidemic? J Med Ethics. 2018;44:343–348. 29146711 10.1136/medethics-2017-104389

[6] General Directorate of Epidemiology . Epidemiological Surveillance System. Zika Surveillance Subsystem. https://www.dge.gob.pe/portal/index.php?option=com_content%26view=article%26id=14%26Itemid=1. Accessed May 17, 2019.

[7] General Directorate of Epidemiology . Epidemiological Surveillance System. Dengue Surveillance Subsystem. http://www.dge.gob.pe/portal/index.php?option=com_content%26view=article%26id=14%26Itemid=121 Accesed May 17, 2019.

[8] Obregón J . Piura: Deaths from dengue already added 41. Peru 21 [website]. 2017 https://peru21.pe/peru/piura-fallecidos-dengue-sumaron-41-390231-noticia/. Accessed May 19, 2019.

[9] Beebe J . Rapid Qualitative Inquiry: A Field Guide to Team‐Based Assessment. Lanham, MD: Rowman & Littlefield Publishers; 2014.

[10] Gray D , Mishtal J . Managing an epidemic: Zika interventions and community responses in Belize. Glob Public Health. 2019;14:9–22. 29733243 10.1080/17441692.2018.1471146

[11] Rasanathan JJ , MacCarthy S , Diniz D , Torreele E , Gruskin S . Engaging human rights in the response to the evolving Zika virus epidemic. Am J Public Health. 2017;107:525–531. 28207337 10.2105/AJPH.2017.303658 PMC5343716

[12] Sharma S , Tyagi A , Ramachandra S , Bhuyan L , Dash KC , Raghuvanshi M . Knowledge, attitudes, and practices among health‐care providers regarding Zika virus infection. J Int Soc Prev Community Dent. 2018;8:41–47. 29629328 10.4103/jispcd.JISPCD_371_17 PMC5853041

[13] Brissett DI , Tuholske C , Allen IE , et al. Zika virus: Knowledge assessment of residents and health‐care providers in Roat an following an outbreak. Am J Trop Med Hyg. 2018;99:211–215. 29761757 10.4269/ajtmh.18-0014 PMC6085802

[14] Quinn C , Poirot E , Sanders Kim A , et al. Variations in healthcare provider use of public health and other information sources by provider type and practice setting during New York City's response to the emerging threat of Zika virus disease, 2016. Health Secur. 2018;16:252–261. 30133373 10.1089/hs.2018.0026

[15] Miranda‐Filho Dde B , Martelli CM , Ximenes RA , et al. Initial description of the presumed congenital Zika syndrome. Am J Public Health. 2016;106:598–600. 26959258 10.2105/AJPH.2016.303115 PMC4816005

[16] Saad T , PennaeCosta AA , de Góes FV , et al. Neurological manifestations of congenital Zika virus infection. Childs Nerv Syst. 2018;34:73–78. 29127595 10.1007/s00381-017-3634-4

[17] Brunoni D , Blascovi‐Assis SM , Osório AA , et al. Microcephaly and other Zika virus‐related events : The impact on children, families and health teams. Cien Saude Colet. 2016;21:3297–3302. 27783802 10.1590/1413-812320152110.16832016

[18] Olórtegui RA , Díaz P . Fetal health and ultrasonographic diagnosis in perinatal Zika virus infection. Peruvian J Gynecol Obstet. 2017;63:71–79.

[19] National Institute of Statistics and Informatics (INEI) . Child Health in Peru: ENDES 2017. Lima: INEI; 2018.

[20] National Institute of Statistics and Informatics (INEI) . Statistical Compendium Piura, 2017. Lima: INEI; 2017.

[21] Valente PK . Zika and reproductive rights in Brazil: Challenge to the right to health. Am J Public Health. 2017;107:1376–1380. 28727526 10.2105/AJPH.2017.303924 PMC5551524

[22] Beare S , Simpson E , Gray K , Andjelic D . Rapid integration of Zika virus prevention within sexual and reproductive health services and beyond: Programmatic lessons from Latin America and the Caribbean. Glob Health Sci Pract. 2019;7:116–127. 30926740 10.9745/GHSP-D-18-00356 PMC6538127

